# Incident psychotic experiences following self-reported use of high-potency cannabis: Results from a longitudinal cohort study

**DOI:** 10.1111/add.16517

**Published:** 2024-05-13

**Authors:** Lindsey A. Hines, Jon Heron, Stanley Zammit

**Affiliations:** 1 Centre for Academic Mental Health, Population Health Sciences, Bristol Medical School, University of Bristol, Bristol, UK; 2 Department of Psychology, University of Bath, Bath, UK; 3 Medical Research Council (MRC) Integrative Epidemiology Unit at the University of Bristol, Bristol, UK; 4 Division of Psychological Medicine and Clinical Neurosciences, MRC Centre for Neuropsychiatric Genetics and Genomics, Cardiff University School of Medicine, Cardiff, UK

**Keywords:** adolescence, ALSPAC, cannabis, psychiatry, psychosis, THC

## Abstract

**Background and aims:**

High-potency cannabis has been associated with increased risk of psychosis, but a lack of prospective data hinders understanding of causality in this relationship. This study aimed to combine prospective report of cannabis use with retrospective report of potency to infer the potency of cannabis used in adolescence and explore whether use of cannabis, and the use of high-potency cannabis, in adolescence is associated with incident psychotic experiences.

**Design:**

Population-based birth cohort study.

**Setting:**

United Kingdom.

**Participants:**

*n* = 5570 participants who reported on any cannabis use (yes/no) age 16 and 18 years, and *n* = 1560 participants from this group who also retrospectively reported on cannabis potency.

**Measurements:**

In questionnaires at ages 16 and 18, individuals self-reported lifetime cannabis use, and at age 24, participants reported the type of cannabis they most commonly used in the whole time since first using cannabis. Psychotic experiences were assessed at age 24 years using the semi-structured Psychosis-Like Symptom Interview, with incident defined as new-onset occurring between ages 19 and 24 years.

**Findings:**

Use of high-potency cannabis at age 16 or 18 was associated with twice the likelihood of experiencing incident psychotic experiences from age 19–24 (Odds Ratio 2.15, 95% Confidence Intervals 1.13–4.06). There was less evidence for an effect of any cannabis use on incident psychotic experiences (Odds Ratio 1.45, 95% Confidence Intervals 0.94–2.12).

**Conclusions:**

Use of high-potency cannabis appears to be associated with increased likelihood of psychotic experiences.

## Introduction

Globally, cannabis is the most commonly used internationally regulated drug [1]. The primary psychoactive component of cannabis is Δ-9-tetrahydrocannabinol (THC), and cannabis with higher concentrations of THC is described as high-potency. Policy liberalization has been accompanied by proliferation of high-potency cannabis in legal markets [2], and THC concentrations have increased in markets where cannabis remains illegal [3].

Adolescent cannabis use is consistently linked to poorer mental health, including psychosis [4]. A recent review of evidence relating to potency identified that use of higher compared to lower potency cannabis was associated with an increased risk of psychosis [5], although determining whether this is causal requires longitudinal data [6].

The authors are unaware of any longitudinal studies with early adolescent measures of psychosis and detail on cannabis potency; however, the Avon Longitudinal Study of Parents and Children (ALSPAC) cohort has prospective measurement of cannabis use, and retrospective self-report of psychotic experiences and potency of cannabis use. We use retrospective report to infer the potency of cannabis used in adolescence and explore whether use of high-potency cannabis in adolescence is associated with incident psychotic experiences.

## Methods

### Study population

**Figure 1:**
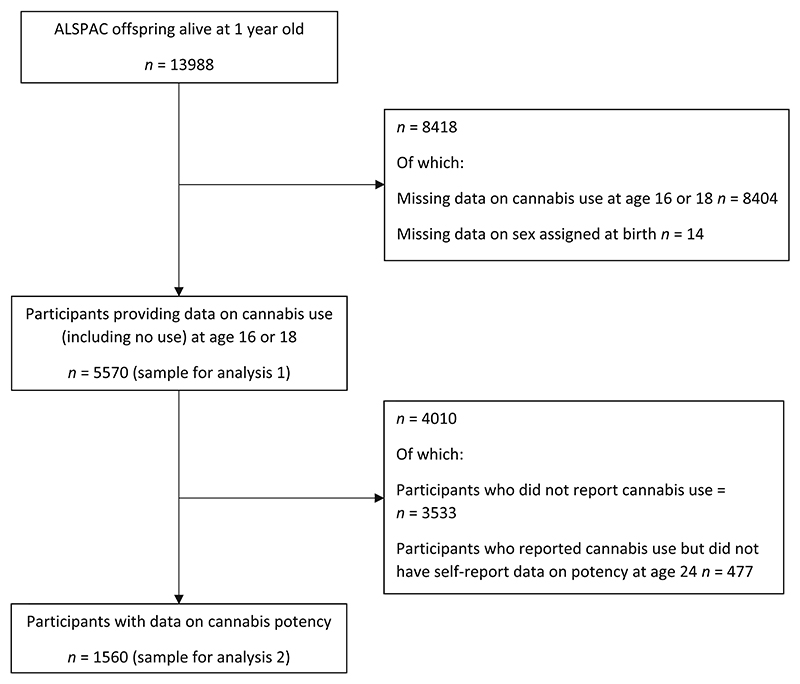
Sample flow diagram.

ALSPAC is a United Kingdom (UK) population-based birth cohort that recruited pregnant women in the former Avon Health Authority with an estimated date of delivery between 1 April 1991 and 31 December 1992; 20 248 pregnancies have been identified as being eligible and the initial number of pregnancies enrolled was 14 541. Of the initial pregnancies, there was a total of 14 676 fetuses, resulting in 14 062 live births and 13 988 children who were alive at 1 year of age. (see Fraser *et al*., Boyd *et al*. and Northstone *et al*. for more detail) [7–9]. Data for the present analyses were collected through self-report postal questionnaire at age 16 and 18, and through self-report questionnaire at an in-person data collection clinic at the University of Bristol at age 24 (travel costs were covered) (see Figure 1).

The present analyses are restricted to participants who reported on cannabis use at either age 16 or 18 (*n* = 5570); for analysis of potency data, this sample was further restricted to those had also reported at age 24 on lifetime type of cannabis most commonly used (*n* = 1560). See Figure 1 for sample derivation.

Please note that the study website contains details of all the data that is available through a fully searchable data dictionary and variable search tool (http://www.bristol.ac.uk/alspac/researchers/our-data/).

### Ethics

All procedures involving human subjects/patients were approved by the ALSPAC Law and Ethics Committee and the Local Research Ethics Committees. Informed consent for the use of data collected via questionnaires and clinics was obtained from participants following the recommendations of the ALSPAC Ethics and Law Committee at the time. Study participation was voluntary, and during data collection information was provided on the intended use of data; consequently returning a questionnaire or attending a clinic was considered written consent. Participants can contact the study team to retrospectively withdraw consent for their data to be used in research at any time.

### Measures

#### Adolescent cannabis use

At ages 16 and 18 individuals self-reported lifetime cannabis use via questionnaire. Responses from these assessments were combined into a binary (yes/no) measure of lifetime adolescent cannabis use. For participants who were missing one wave, the response at the available wave was used.

#### Adolescent cannabis potency

It is important to note that cannabis potency was not measured concurrently with reported cannabis use age 16/18 years and was self-reported for the first time at age 24. This measure is being used to infer the likely type of cannabis used during adolescence. Participants self-reported the type of cannabis they most commonly used in the whole time since first using cannabis (options: ‘herbal cannabis/marijuana’, ‘skunk/other stronger types of herbal cannabis’, ‘hashish/resin/solid’, ‘other’ or ‘do not know’) via computer questionnaire at the data collection clinic. Consistent with previous research that has validated self-reported data on these cannabis types against quantified concentrations of THC and cannabidiol among young UK cannabis users [10], we categorized the most commonly used cannabis as either high-potency (typically ≥10% THC; ‘skunk/other stronger types of herbal cannabis’) or low-potency (typically <10% THC; ‘herbal cannabis/marijuana’ or ‘hashish/resin/solid’ or ‘other’). This measure was combined with the measure of adolescent lifetime cannabis use to generate a variable inferring ‘adolescent low-potency cannabis use’ (cannabis use by age 18, and most commonly used cannabis type reported at age 24 was low-potency), and ‘adolescent high-potency cannabis use’ (cannabis use by age 18, and most commonly used cannabis type reported at age 24 was high-potency).

#### Psychosis outcomes

Psychotic experiences were assessed at age 24 years using the semi-structured Psychosis-Like Symptom Interview (PLIKSi), which elicits information on 12 key psychotic experiences: hallucinations (visual and auditory), delusions (spied on, persecution, thoughts read, reference, control, grandiosity and other) and experiences of thought interference (broadcasting, insertion and withdrawal). Coding followed glossary definitions and rating rules for the Schedules for Clinical Assessment in Neuropsychiatry (SCAN). SCAN-trained psychology graduates rated psychotic experiences as not present, suspected or definitely present (only rated as definite when an example that clearly met SCAN rating rules was provided). The PLIKSi shows good reliability. [11] Participants were asked to report on psychotic experiences since age 12, and if these were rated as meeting criteria for a psychotic experience, they were then asked when it first happened. Incident was defined as new-onset (occurring between ages 19 and 24 years) for: (1) definite psychotic experiences; and (2) definite psychotic experiences that were either frequently recurring (≥monthly) or rated as quite or very distressing. Individuals who met psychosis criteria before age 19 were included in the ‘no incident psychotic experience’ comparator group.

#### Confounders

Childhood socio-economic position (SEP) (maternal educational attainment, and parents’ occupation class) was assessed from maternal questionnaires completed during pregnancy, and tobacco and alcohol use from questionnaires at ages 16 and 18, with participants reporting any use of tobacco or alcohol included as cases. Symptoms of depression were assessed at age 16 using the short mood and feelings questionnaire [12]. Participant sex was assessed at birth, and was a binary male/female variable.

### Analysis

Analyses were conducted in Stata version 16.1. The analysis was not pre-registered and the results should be considered exploratory.

Univariable and multivariable logistic regression, with effect estimates presented as odds ratio (OR) and 95% confidence intervals (CI), were used to assess the relationship between adolescent cannabis use and incident psychotic experiences. Two separate models were used to allow comparison of the effects of any cannabis use on incident psychotic experiences relative to no use, and the effects of high-potency cannabis use relative to lower-potency cannabis use. The models were (1) associations between cannabis exposure at age 16/18 and incident psychotic experiences after age 19 (among all participants who reported on cannabis use at age 16/18, including those reporting no use); and (2) associations between inferred cannabis potency aged 16/18 (informed by lifetime cannabis potency, self-reported at age 24) and incident psychotic experiences after age 19 (only among those reporting cannabis use age 16/18).

Missing data in outcomes and covariates (but not exposures) were addressed through multiple imputation using chained equations, a series of regression models that impute each incomplete variable sequentially. Data were assumed to be missing at random. Separate models were run for the exposure of any cannabis use and for cannabis potency (removing those who did not report cannabis use and did not have potency data), as these formed two separate samples. Forty datasets were generated for the any cannabis use sample, and 40 imputed datasets for the cannabis potency sample, guided by results of Monte Carlo error estimates. Estimates were obtained using Rubin’s rules^14^ by pooling results across the 40 datasets. Missing data patterns are reported in Tables S1 and S2.

## Results

### Sample description

Of the 5570 people who reported on cannabis use (including reporting no use) age 16 to 18, 2037 (36.6%) reported having ever used cannabis. See Table S3 for sample description. Participants who reported cannabis use were similar to those reporting no cannabis use on sex, SEP, alcohol use and depression scores, but tobacco use was higher among those reporting cannabis use. Of those for whom potency data were available (*n* = 1560, see Figure 1), 145 (9.3%) reported use of high-potency cannabis. Those using high-potency cannabis were similar to those using lower-potency cannabis on alcohol use and depression scores, but were more likely to be male and to use tobacco, and less likely to have parents with lower occupational class.

### Association with incident psychosis

Incident psychotic experiences were reported by 6.4% of adolescents who used cannabis, compared to 3.8% of those who did not report cannabis use. Incident frequent/distressing incident psychotic experiences were reported by 2.6% of adolescents who used cannabis, compared to 1.8% of those who did not report cannabis use.

In both adjusted and unadjusted analyses, there was only weak evidence adolescents who used cannabis had greater likelihood of incident psychotic experiences.

Among those for whom we have inferred use of high-potency cannabis at age 16 to 18, incident psychotic experiences were reported by 10.1% compared to 4.5% of those using lower-potency cannabis. Incident frequent/distressing incident psychotic experiences were reported by 4.3% of adolescents who used high-potency cannabis, compared to 1.8% of those using lower-potency cannabis.

Relative to those who were likely using lower-potency cannabis, those using high-potency cannabis were twice as likely to experience an incident psychotic experience (adjusted OR [AOR] = 2.38, 95% CI = 1.30–4.38), and there was weak evidence they more than twice as likely to have incident psychotic experiences that were frequent or distressing (AOR = 2.43, 95% CI = 0.92–6.42). See Table 1 for results and Table S4 for results in complete case data.

## Discussion

In lieu of prospective data on cannabis potency we have inferred use of high-potency cannabis during adolescence; by combining this with psychosis outcomes that were incident after the selected period of cannabis use, this study suggests that high-potency cannabis use may increase likelihood of psychotic experiences more than low-potency use, although numbers with frequent or distressing experiences were small and estimates imprecise. It is important to note that cannabis potency was not measured concurrently with reported cannabis use age 16/18 years, and was self-reported for the first time at age 24. This measure is being used to infer the likely type of cannabis used during adolescence.

The present work contributes to a mixed picture on the relationship between use of high-potency cannabis and psychosis. In a case–control study using a clinical sample, those who used high-potency cannabis were at greater risk of psychosis compared to people who never used cannabis [13], whereas cross-sectional analyses from the ALSPAC cohort found weak associations between use of high-potency cannabis and incident psychotic experiences when comparing high- and low-potency cannabis use [6]. Analyses of health registries in Denmark has indicated that the incidence of psychosis has risen as cannabis potency has increased [14], but no previous work has linked cannabis potency and incidence of psychosis in individuals.

As the potency analyses are restricted to those who use cannabis, both groups are similar on many characteristics. It is widely report in the literature that people who use cannabis differ from those who do not on risk factors such as genes, adversity exposure, mental health status and tobacco use. By comparing potency among people who use cannabis, we attempted to hold much of that variation constant and, therefore, isolate the effect of potency from unmeasured confounding.

### Limitations

This study is the first to include temporality in the relationship between cannabis potency and psychosis, but there are several limitations. In an illegal market, there is no verified information provided on the potency of cannabis, and consequently we cannot be certain participants are correct when stating the type of cannabis that they are using. Second, because of attrition within ALSPAC those who took part in the adolescent data collection at the age 24 wave of the study were more likely to be white, female and more affluent than the area population which they were recruited from [8]. As outcome data were collected at age 24 (compared to exposure data being collected age 16/18), there was large amounts of missing data on these variables. There are no large differences in the characteristics of those who did and did not have data on the outcome (see Table S5) or on potency data (see Table S6). The analyses would benefit from replication in larger, representative samples with contemporaneous measures, but we are unaware of any longitudinal cohorts that include measures of cannabis potency and validated measures of psychosis. Third, the measure of incident psychotic experiences used in the analysis did not distinguish psychotic experiences that occurred as a result of cannabis use; consequently some of the variation in the results may be attributable to this. Fourth, as potency was not measured concurrently with cannabis use in adolescence it is plausible that psychosis onset did precede the period in which high-potency cannabis began to be used. Until prospective data are collected on cannabis potency and psychosis, this issue will remain unresolved.

### Implications

This work contributes to a growing body of evidence indicating that use of high-potency cannabis is associated with increased likelihood, and (in the present case) incidence, of psychotic experiences. Measures to reduce the potency of cannabis in both regulated and unregulated cannabis markets should be explored.

## Supplementary Material

Supplement

## Figures and Tables

**Table 1:** Association between any cannabis use at age 16–18, and inferred potency of cannabis used age 16–18, and incident psychosis outcomes age 19–24.

*n* = 5570	Incident psychotic experience age 19–24, *n* = 265^a^			Incident frequent or distressing psychotic experience age 19–24, *n* = 122^a^	
	Univariable OR (95% CI), P value	Multivariable^b^ OR (95% CI), P Value		Univariable OR (95% CI), P value	Multivariable^b^ OR (95% CI), P value
Adolescent cannabis use, *n* = 2037	1.80 (1.22–2.64), 0.003	1.45 (0.94–2.12), 0.092		1.57 (0.98–2.51), 0.059	1.01 (0.58–1.78), 0.960
No adolescent cannabis use, *n* = 3533	1.0	1.0		1.0	1.0
	**Incident psychotic experience age 19–24, *n* = 78** ^a^			**Incident frequent or distressing psychotic experience** **age 19–24, *n* = 32** ^a^	
***n* = 1560**	**Univariable OR (95% CI),** **P value**	**Multivariable**^c^ **OR (95% CI)**, **P value**		**Univariable OR (95% CI),** **P value**	**Multivariable**^c^ **OR (95% CI),** **P value**
Adolescent high-potency cannabis use (inferred), *n* = 145	2.38 (1.30–4.38), 0.005	2.15 (1.13–4.06), 0.019		2.45 (0.99–6.10), 0.054	2.43 (0.92–6.42), 0.074
Adolescent lower-potency cannabis use (inferred), *n* = 1415	1.0	1.0		1.0	1.0

a Numbers derived from imputed proportions.

b Adjusted for tobacco use age 16–18, alcohol use age 16–18, social class, maternal education, sex and depression symptoms at age 16.

c Adjusted for tobacco use age 16–18, social class, maternal education, sex and depression symptoms at age 16. Alcohol was dropped from these models because of lack of variation between exposure and outcome groups.

## Data Availability

The informed consent obtained from ALSPAC participants does not allow for the data to be made freely available through any third party maintained public repository. However, data used for this Article can be made available on request to the ALSPAC Executive. The ALSPAC data management plan describes in detail the policy regarding data sharing, which is through a system of managed open access. Full instructions for applying for data access can be found here: http://www.bristol.ac.uk/alspac/researchers/access/. The ALSPAC study website contains details of all the data that are available (http://www.bristol.ac.uk/alspac/researchers/our-data/).
