# Supplementary material for: Incident psychotic experiences following self-reported use of high-potency cannabis: Results from a longitudinal cohort study
Source: Addiction. Author manuscript; Available in PMC 2024 Nov 25. (PMC7616847; doi:10.1111/add.16517)
Supplement: Supplement [file EMS200188-supplement-Supplement.docx]

**Appendix**

| **Page** | **Content** |
| --- | --- |
| 2 | Supplementary Table 1: Proportion of missing data in the sample restricted to participants with data available on any cannabis use (n=5570) |
| 3 | Supplementary Table 2: Proportion of missing data in the sample restricted to participants with data available on cannabis potency (n=1560) |
| 4 | Supplementary Table 3: Sample characteristics in imputed data |
| 5 | Supplementary Table 4: Complete case analyses of the association between any cannabis use at age 16-18, and inferred potency of cannabis used age 16-18, and incident psychosis outcomes age 19-24 |

Supplementary Table 1: Proportion of missing data in the sample restricted to participants with data available on any cannabis use (n=5570)

|  | Proportion missing data | Prevalence/mean in complete case data | Prevalence/mean in imputed dataset |
| --- | --- | --- | --- |
| Incident psychotic experience age 19-24 | 47.49 | 4.38 | 4.76 |
| Incident frequent or distressing psychotic experience age 19-24 | 47.49 | 1.95 | 2.19 |
| Sex (female) | 0 | 59.68 | - |
| Tobacco use age 16-18 | 24.13 | 69.43 | 65.01 |
| Alcohol use age 16-18 | 3.21 | 98.48 | 98.41 |
| Social class (skilled/unskilled occupations) | 10.88 | 35.54 | 35.96 |
| Maternal education (lower) | 7.04 | 19.08 | 19.26 |
| Depression symptoms at age 16 | 10.97 | 5.64 | 5.66 |

Supplementary Table 2: Proportion of missing data in the sample restricted to participants with data available on cannabis potency (n=1560)

|  | Proportion missing data | Prevalence/mean in complete case data | Prevalence/mean in imputed dataset  (N=1560) |
| --- | --- | --- | --- |
| Incident psychotic experience age 19-24 | 2.63 | 5.00 | 5.02 |
| Incident frequent or distressing psychotic experience age 19-24 | 2.63 | 2.04 | 2.07 |
| Sex (female) | 0 | 63.59 | - |
| Tobacco use age 16-18 | 12.24 | 83.27 | 82.85 |
| Social class (skilled/unskilled occupations) | 9.74 | 29.62 | 29.92 |
| Maternal education (lower) | 6.22 | 12.71 | 12.95 |
| Depression symptoms at age 16 | 9.04 | 6.34 | 6.38 |

Supplementary Table 3: Sample characteristics in imputed data

|  | Adolescent cannabis use  N=5570 | | Adolescent cannabis potency  N=1560 | |
| --- | --- | --- | --- | --- |
|  | Cannabis use  N=2037  %  (95% CI) | No cannabis use  N=3533  %  (95% CI) | High-potency cannabis use  N=145  %  (95% CI) | Lower-potency cannabis use  N=1415  %  (95% CI) |
| Incident psychotic experience age 19-24 | 6.54%  (4.93% - 81.60%) | 3.74%  (2.79% - 4.69%) | 10.10%  (5.08% -15.12%) | 4.50%  (3.41%-5.59%) |
| Incident frequent or distressing psychotic experience age 19-24 | 2.83%  (1.86% - 3.80%) | 1.82%  (1.16% - 2.48%) | 4.40%  (0.93% - 7.86%) | 1.83%  (1.12%-2.54%) |
| Sex (female) | 61.81%  (59.70% - 63.92%) | 58.45%  (56.82% - 60.07%) | 35.17%  (27.39% - 42.95%) | 66.50%  (64.04%-68.96%) |
| Social class (skilled/unskilled occupations) | 34.74%  (32.55% - 36.94%) | 36.66%  (34.99% - 38.34%) | 23.26%  (16.00% - 30.52%) | 30.61%  (28.11%-33.12%) |
| Maternal education (lower) | 17.88%  (16.13% - 19.63%) | 20.06%  (18.68% -21.43%) | 12.95%  (7.22% - 18.68%) | 12.95%  (11.15%-14.76%) |
| Tobacco use age 16-18 | 93.94%  (92.88% - 95.00%) | 48.24%  (46.33% - 50.15%) | 93.50%  (89.18%-97.82%) | 81.75%  (79.63%-83.87%) |
| Alcohol use age 16-18 | 99.95%  (99.85%- 100.05%) | 97.52%  (96.98% - 98.06%) | 100.00%  (100.00%-100.00%) | 99.64%  (99.33%-99.96%) |
| Depression symptoms at age 16 (mean) | 6.90  (6.63 -7.17) | 5.39  (5.21 - 5.58) | 6.56  (5.58-7.55) | 6.36  (6.06-6.66) |

Supplementary Table 4: Complete case analyses of the association between any cannabis use at age 16-18, and inferred potency of cannabis used age 16-18, and incident psychosis outcomes age 19-24

|  | Incident psychotic experience age 19-24  **N=128** | | Incident frequent or distressing psychotic experience age 19-24  **N=57** | |
| --- | --- | --- | --- | --- |
|  | Univariable  OR (95%CI) | Multivariable^1^  OR (95%CI) | Univariable  OR (95%CI) | Multivariable^1^  OR (95%CI) |
| Cannabis use age 16-18 | 1.87  (1.09 – 3.25) | 2.33  (0.95 – 5.75) | 1.51  (0.89 – 2.55) | 1.37  (0.58 – 3.20) |
| No cannabis use age 16-18 | 1.0 | 1.0 | 1.0 | 1.0 |
|  | | | | |
|  | Incident psychotic experience age 19-24  **N=76** | | Incident frequent or distressing psychotic experience age 19-24  **N=31** | |
|  | Univariable  OR (95%CI) | Multivariable^2^  OR (95%CI) | Univariable  OR (95%CI) | Multivariable^2^  OR (95%CI) |
| High-potency cannabis use age 16-18 | 2.36  (1.28 – 4.34) | 2.63  (1.26 – 5.49) | 2.42  (0.98 – 6.02) | 1.99  (0.62 – 6.35) |
| Lower-potency cannabis use age 16-18 | 1.0 | 1.0 | 1.0 | 1.0 |

^1^Adjusted for tobacco use age 16-18, alcohol use age 16-18, social class, maternal education, sex, and depression symptoms at age 16

^2^Adjusted for tobacco use age 16-18, social class, maternal education, sex, and depression symptoms at age 16

Supplementary table 5: Characteristics of missing outcome data in the sample restricted to participants with data available on any cannabis use (n=5570)

|  | Outcome data available^1^ | Outcome data missing^2^ |
| --- | --- | --- |
| Cannabis use age 16-18 | 35.50% | 37.54% |
| Tobacco use age 16-18 | 64.07% | 76.46% |
| Female | 65.3% | 53.46% |
| Higher social class | 68.26% | 60.15% |
| Higher maternal education | 85.6% | 75.6% |

^1^Participants who provided data for both incident psychotic experience age 19-24 variable, and incident frequent or distressing psychotic experience age 19-24.

^2^Any participant missing on Incident psychotic experience age 19-24 variable, or Incident frequent or distressing psychotic experience age 19-24.

Supplementary table 6: Characteristics of missing potency data amongst those who reported using cannabis age 16/18 (N=2037)

|  | Potency data available | Potency data missing |
| --- | --- | --- |
| Tobacco use age 16-18 | 83.2% | 95.9% |
| Female | 63.6% | 58.5% |
| Higher social class | 70.4% | 60.8% |
| Higher maternal education | 87.3% | 78.2% |
